# The Effectiveness of a Preoperative Check-and-Consent Clinic in Reducing Day-of-Surgery Cancellations in Elective Hand Surgery: A Retrospective Service Evaluation

**DOI:** 10.7759/cureus.101931

**Published:** 2026-01-20

**Authors:** Yahya Abu-Seido, Ahmed Ali, Zaid Haj Ali, Safina Begum, Frances Freer, James Li, Michael David

**Affiliations:** 1 Trauma and Orthopaedics, University Hospitals Coventry and Warwickshire (UHCW) NHS Trust, Coventry, GBR; 2 Trauma and Orthopaedics, University of Manchester, Manchester, GBR; 3 Trauma and Orthopaedics, Addenbrooke's Hospital, Cambridge University Hospitals NHS Foundation Trust, Cambridge, USA; 4 Plastic Surgery, University Hospitals Coventry and Warwickshire (UHCW) NHS Trust, Coventry, GBR; 5 Trauma and Orthopaedics, University College London Hospitals (UCLH) NHS Foundation Trust, London, GBR; 6 Orthopaedic Surgery, University Hospitals Coventry and Warwickshire (UHCW) NHS Trust, Coventry, GBR

**Keywords:** cost-efficiency, day case surgery, elective procedures, pre-operative planning, surgery cancellations, surgical informed consent, value-based healthcare

## Abstract

Day-of-surgery cancellations (DoSC) waste resources, increase costs, and cause patient dissatisfaction. They are common and often preventable. With growing reliance on allied healthcare professionals and virtual clinics, patients can be listed without surgeon review or a Montgomery-compliant consent process, leading to last-minute changes. A surgeon-led "check-and-consent" clinic was introduced to reduce potential DoSC in elective hand surgery.

A retrospective review was conducted of patients scheduled for elective day-case hand surgery and seen in the check-and-consent clinic (April 2022-January 2024). A consultant surgeon reassessed symptoms, reconfirmed consent, adjusted management as required, and obtained written consent. Primary outcomes were management changes and potential DoSCs avoided. Secondary outcomes included cost impact using National Health Service (NHS) tariffs.

Sixty-seven patients were scheduled; one did not attend, leaving 66 analyzed (mean age, 58.1 years). Carpal tunnel syndrome was the most common diagnosis (77.3%). Management changed in 29 patients (43.9%): 12 were converted to nonoperative care, eight declined or did not require treatment, and nine had a modified surgical plan. A minimum of 20 DoSCs (30.3%) were avoided. Cost analysis demonstrated net financial savings after accounting for clinic costs.

A dedicated check-and-consent clinic reduced avoidable DoSCs, improved theater efficiency, and reinforced the principle of shared decision-making. This low-cost intervention offers measurable clinical and economic benefits and could be adopted in other elective surgical pathways.

## Introduction

Day-of-surgery cancellations (DoSC) are a persistent problem in healthcare systems, reducing efficiency, increasing costs, and causing distress to patients [1-3]. Reported rates vary widely, from 2% to 24% across institutions and specialties [4]. Importantly, over 80% of cancellations are due to preventable administrative or patient-related factors rather than unforeseen clinical issues [5,6]. These include patients becoming medically unfit, symptom resolution, changes in decision, or failure to attend (did not attend (DNA)) [7]. This issue is especially relevant in elective hand surgery, where conditions such as carpal tunnel syndrome, trigger finger, and ganglia may fluctuate during long waiting periods and often respond to nonoperative interventions like steroid injections or splinting [8]. National Health Service (NHS) England reported 22,681 last-minute cancellations in Q3 2024/2025, representing 1.0% of all elective operations; concerningly, 23% were not rescheduled within 28 days, a steep rise from 7% in 2015/2016 [9]. Such delays waste capacity and undermine surgical throughput.

The problem has been compounded by increasing demand on NHS services, with growing reliance on allied health professionals (e.g., extended-scope physiotherapists and nurse specialists), as well as virtual consultations. While this model is efficient and cost-effective, it carries risks: some patients may be listed for an inappropriate procedure or may not have undergone a Montgomery-compliant consent process, only to meet the surgeon for the first time on the day of surgery. In other cases, a different diagnosis may be uncovered at this late stage. Both scenarios contribute to avoidable DoSC, which are costly for the NHS and distressing for patients. To address this, the University Hospitals Coventry and Warwickshire (UHCW) Hand Unit implemented a surgeon-led “check-and-consent” clinic, ensuring all patients are reviewed by a consultant surgeon before surgery. Conventional preoperative assessment clinics have reduced cancellations by optimizing medical fitness, but these are usually nurse-led and rarely reappraise the indication for surgery [10,11]. Evidence from urology shows that structured reassessment can cut cancellations dramatically, with a specialist pre-assessment clinic reducing rates from 23.9% to 4.3% [10]. NICE also advocates early identification of high-risk patients to support shared decision-making and improve perioperative outcomes [11]. Consent must similarly be treated as an ongoing process. The General Medical Council (GMC) and Royal College of Surgeons emphasize that valid consent must be informed, up-to-date, and responsive to changes in patient preferences or circumstances [12-14]. To operationalize these standards, the consultant-led “check-and-consent” clinic was designed to reassess symptoms, review comorbidities, and reconfirm consent shortly before surgery.

This study was undertaken to evaluate the role of the check-and-consent clinic within our elective hand surgery pathway. The clinic provides a focused, consultant-led reassessment for patients who were either listed without a recent face-to-face review or had been waiting several months since their last consultation. Its purpose is to confirm the ongoing indication for surgery, review any change in symptoms or preferences, and ensure that consent discussions are clear and up-to-date. The specific aims of the study were to describe how often management plans changed at this clinic, estimate the number of potential DoSC that may have been avoided, and assess the financial impact of reviewing patients in this way.

## Materials and methods

Research methodology

A retrospective observational study was conducted at a single tertiary hand unit within an NHS trust to evaluate the effectiveness of a surgeon-led check-and-consent clinic in reducing DoSC for elective day-case hand surgery. Eligibility criteria included all consecutive patients scheduled to undergo elective hand surgery who either (1) had been listed without a face-to-face review by a consultant hand surgeon within the preceding six weeks or (2) had been waiting longer than six months since their last face-to-face review. All eligible patients were invited to attend a surgeon-led check-and-consent clinic between April 14, 2022, and January 18, 2024, via phone call, SMS, and letters posted to their home address. Patients who did not attend were excluded from the main analysis. As this was a service evaluation using fully anonymized data, formal research ethics committee approval was not required under UK Health Research Authority (HRA) guidelines. The six-week and six-month cut-offs were based on local practice. After six weeks without a consultant review, symptoms or patient preferences may have changed, and a brief reassessment is often helpful. The six-month threshold was used for patients previously seen face-to-face, as this was felt to be a reasonable point at which the original assessment might need updating.

The intervention: check-and-consent clinic

The clinic was conducted by a single consultant orthopedic hand surgeon and served as a final preoperative review, typically occurring weeks before the scheduled surgery date. Each consultation included a full clinical reassessment of the patient’s symptoms and functional status, a review of comorbidities and assessment of fitness for surgery, and an explicit informed consent process, aligned with the GMC and the Royal College of Surgeons (RCS) guidance on supported decision-making. This involved the completion and signing of a paper consent form, thereby avoiding reliance on signatures obtained on the day of surgery, which may be legally challenged as coerced consent. Consent was, however, reconfirmed on the day of surgery to ensure contemporaneous validity [13,15]. This did not replace the need for consent on the day of surgery; rather, it provided an earlier and more detailed discussion. Based on this review, the management plan was either confirmed or altered. Changes included proceeding with the planned surgery, modifying the surgical procedure (e.g., change of technique or laterality), or abandoning surgery in favor of nonoperative management.

Classification of management changes

Management changes were determined by comparing the initial management plan with the definitive management documented in clinic letters, with all free-text entries coded into one of three mutually exclusive categories using a standardized coding dictionary. The first category, no treatment (surgery abandoned/DoSC avoided), included cases where the clinic explicitly recorded cancellation, abandonment, discharge, onward referral, or the patient becoming unfit for surgery, such as “cancelled - spontaneous resolution” or “potential alternative diagnosis needs further investigation.” The second category, nonoperative management (converted to conservative care), captured situations where surgery was deferred in favor of conservative measures like injections, splinting, physiotherapy, or trials of nonsurgical care, for example, “postponed - trial of steroid injection and night splinting.” The final category, modified surgical plan, encompassed cases where surgery proceeded, but the intended procedure was altered, including changes in laterality, staging, surgical technique, or specific operation, such as “changed from open unilateral to bilateral endoscopic carpal tunnel release.” Although some of these modifications could theoretically avert a potential DoSC (such as when equipment shortages necessitate procedural changes), they were excluded from the cost analysis because surgery could still reasonably proceed on the planned date. To ensure each case was assigned a single category, hierarchical rules were applied (no treatment > nonoperative > modified), and duplicate patient entries were avoided by retaining only the most recent clinic note. The complete coding dictionary with example terms is shown in Appendix 1, and examples of redacted clinic notes demonstrating how classifications were applied are provided in Appendix 2.

Data collection and analysis

Data were extracted retrospectively from electronic patient records, including clinic letters and operative notes. The following variables were collected for each patient: demographics (age and gender), source of referral, primary diagnosis, original planned procedure, final management decision following the check-and-consent clinic, reason for any change in plan, and operation date (if surgery proceeded). Further information on dataset availability is included in the Appendix section "Anonymized dataset availability." Descriptive statistics were used to summarize patient demographics and clinic outcomes. To support reproducibility, we have clarified several elements of the study process. The check-and-consent clinic operated as a standard consultant-led review within existing job plan time, and no additional staff were appointed for this service. Coding of management changes was performed by the study author using a predefined dictionary, with uncertain cases reviewed by a consultant surgeon to ensure consistency. Missing data were minimal and handled by excluding variables that were not documented in the clinic letters or records; missing information in individual records was left as missing, and no attempts were made to replace or estimate these values. As this was a service evaluation, the focus was on describing outcomes rather than validating inter-rater reliability, but the classification approach is described in clear terms to support replicability. Continuous variables are presented as mean ± standard deviation (SD) or median (range), and categorical variables are presented as counts and percentages. For proportions, 95% confidence intervals (CIs) were calculated using the Wilson method for binomial data. Statistical analysis was performed using Microsoft Excel (Microsoft Corp., Redmond, WA). No inferential statistical tests (t-tests, χ² tests, and ANOVA) were performed because this was a descriptive service evaluation. Only descriptive statistics (n, %, mean ± SD, median, and range) are reported.

## Results

Patient characteristics

A total of 67 patients were scheduled to attend the check-and-consent clinic, of whom 66 attended during the study period. The mean age of the cohort was 58.1 years (± 15.3, range: 17-85), with 34 males (51.5%) and 32 females (48.5%). The most common diagnosis was carpal tunnel syndrome (CTS), present in 51 patients (77.3%), either unilaterally or bilaterally, including two cases with overlapping cubital tunnel syndrome. Other diagnoses included Dupuytren's disease (9.1%), trigger finger (4.5%), and ganglion (3.0%). The median waiting time from listing to clinic attendance was 78.5 days (range: 0-514).

Eligibility for the check-and-consent clinic included patients listed for elective hand surgery who had not been reviewed face-to-face by a consultant hand surgeon in the previous six months or those whose listing had been made by an allied health professional. Most patients (80.3%) were referred by hospital-based clinicians, and the remainder were referred from primary care (18.2%) or a consultant-led satellite clinic (1.5%). The diagnoses are summarized in Table 1.

**Table 1:** Primary diagnosis breakdown of patients attending the check-and-consent clinic (n = 66) Data presented as n (%), mean ± SD, or median (range). No statistical tests were performed. Significance thresholds (if applicable): p < 0.05, p < 0.001.

Primary diagnosis	n	%
Carpal tunnel syndrome (CTS)	51	77.3
Dupuytren’s disease	6	9.1
Trigger finger	3	4.5
Ganglion	2	3.0
Other	4	6.1

Cost analysis

Cost estimates were based on the NHS payment by results tariff system, which assigns a nationally set reimbursement price (tariff) to episodes of care according to Healthcare Resource Group (HRG) codes. In this analysis, the cost of a first outpatient attendance (£169) was applied to each attended check-and-consent clinic visit. The cost of a DNA appointment was not included, as DNAs do not generate a tariff reimbursement. For surgical costs, the 2022/23 NHS tariff for intermediate day-case hand procedures was applied (HRG code HB21C-D, approximately £1,140) [16].

The net financial impact was calculated as:

$(Number\ of\ avoided\ procedures\ &times;\ surgical\ tariff) &minus; (Number\ of\ attended\ clinic\ visits\ &times;\ clinic\ tariff).$

In the base-case scenario, a minimum of 20 procedures were avoided among 66 attendees, resulting in an estimated saving of £22,800 against a clinic cost of £11,154. The net saving was therefore £11,646. If DNA slots were also costed (67 total clinic slots), the net saving remained similar at £11,477 (Table 2). To ensure robustness, a sensitivity analysis was performed, varying surgical tariffs by ±10% and including or excluding DNA clinic slot costs. The net financial benefit ranged from £9,366 to £13,926, demonstrating that the intervention remained cost-saving under all plausible assumptions. This clinic did not require additional staffing or new resources; it was delivered within the existing consultant job plan time and replaced a portion of routine outpatient activity. The potential savings arise from identifying patients who no longer need surgery, thereby avoiding the theater costs associated with procedures that would otherwise proceed. In this sense, the clinic functions as an additional safety check within the current pathway rather than as a separate or expanded service.

**Table 2:** Cost analysis of the check-and-consent clinic Data presented as n (%), mean ± SD, or median (range). No statistical tests were performed. Significance thresholds (if applicable): p < 0.05, p < 0.001.

Scenario	Clinic cost (£)	Avoided surgery cost (£)	Net saving (£)
Base case (66 attended, 20 avoided)	11,154	22,800	11,646
Include DNA slot cost (67 total)	11,323	22,800	11,477
Higher surgical tariff (+10%)	11,154	25,080	13,926
Lower surgical tariff (−10%)	11,154	20,520	9,366

Clinical outcomes and avoided cancellations

Of the 66 patients who attended the check-and-consent clinic, the management plan was altered for 29 (43.9%). These changes fell into three mutually exclusive categories (Table 3): conversion to nonoperative management, no treatment/cancelled surgery, and finally modification of the planned surgical procedure. About 12/66 (18.2%, 95% CI: 10.7-29.1) were converted to nonoperative management, and patients were moved off the planned surgical pathway in favor of conservative care. The conservative strategies recorded included corticosteroid injection (seven patients), trial of splinting (four patients), and one case converted to another nonoperative approach (documented as “nonoperative” without further specification). Surgery was recorded as cancelled or not presently required for eight patients (8/66 (12.1%, 95% CI: 6.3-22.1)) for various reasons documented in the clinic notes, including spontaneous resolution of symptoms, patient choice to decline surgery, transfer of care to another surgeon, and medical unfitness. Lastly, 9/66 (13.6%, 95% CI: 7.3-23.9) required modification of the planned surgical procedure; specific changes included unilateral to bilateral carpal tunnel release, bilateral to unilateral procedures, bilateral procedures changed to staged unilateral operations, adjustments in laterality (side of surgery), and modifications to Dupuytren’s surgical technique.

**Table 3:** Outcomes for patients attending the check-and-consent clinic (n = 66) Data presented as n (%), mean ± SD, or median (range). No statistical tests were performed. Significance thresholds (if applicable): p < 0.05, p < 0.001.

Outcome category	n	%	95% CI
Management unchanged	37	56.1	44.1–67.4
Nonoperative management	12	18.2	10.7–29.1
No treatment	8	12.1	6.3–22.1
Modified surgical plan	9	13.6	7.3–23.9
Planned surgeries avoided	20	30.3	20.6–42.2

**Figure 1:**
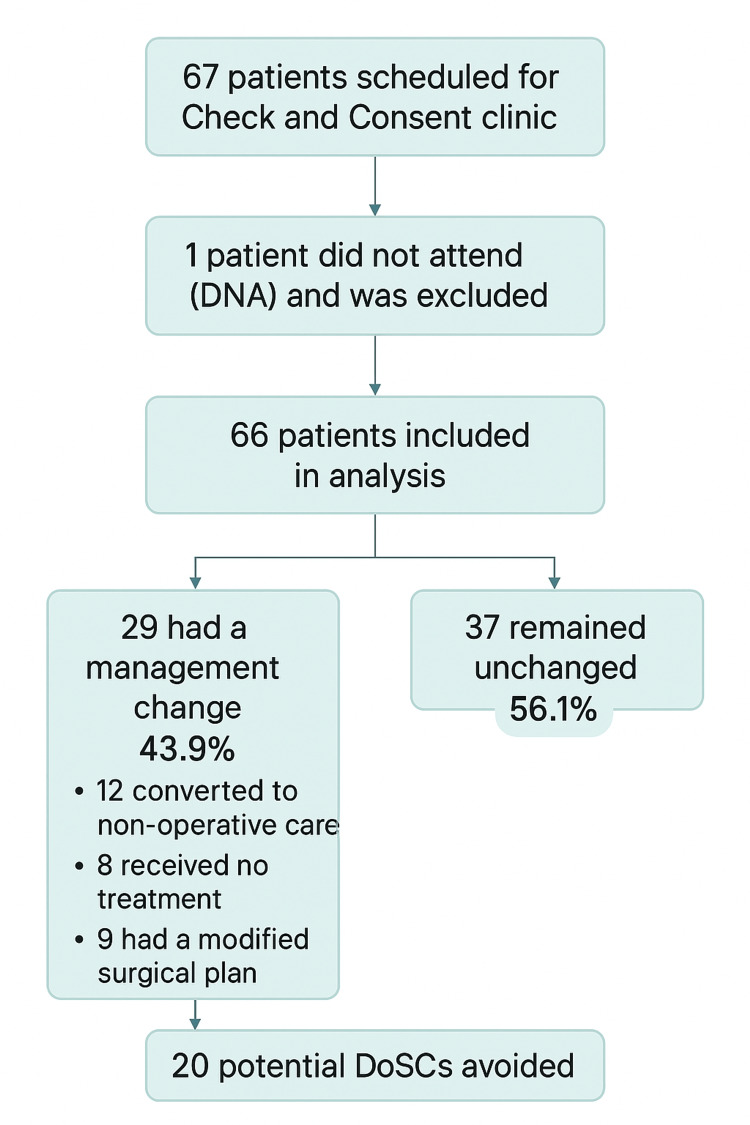
STROBE flow diagram of patient inclusion and outcomes at the check-and-consent clinic Sixty-seven patients were scheduled for the check-and-consent clinic. One patient did not attend (DNA) and was excluded, leaving 66 patients included in the analysis. Of these, 29 (43.9%) had a management change, 12 (18.2%) were converted to nonoperative care, eight (12.1%) received no treatment, and nine (13.6%) had a modified surgical plan, while 37 (56.1%) remained unchanged. A total of 20 (30.3%) potential day-of-surgery cancellations (DoSC) were avoided. Data were presented as n (%). No statistical tests were performed. Significance thresholds (if applicable): p < 0.05, p < 0.001. STROBE: Strengthening the Reporting of Observational Studies in Epidemiology.

Consequently, a minimum of 20 patients (30.3% of attendees) who would likely have been cancelled on the day of surgery were identified proactively in the clinic. One additional patient did not attend the clinic and subsequently had their surgery cancelled, but this case was excluded from the main analysis. The patient who did not attend was contacted through the usual trust process and offered a new appointment where possible. Therefore, the total number of potential DoSCs avoided within the analyzed cohort was 20. The overall flow of patient inclusion and outcomes is illustrated in Figure 1.

Follow-up outcomes

All patients were tracked for downstream outcomes after their check-and-consent clinic appointment. Several patients who initially deferred surgery in favor of injection subsequently underwent successful carpal tunnel release within 12 months. In some cases, injections provided temporary relief, but recurrence prompted later surgery, while in others, they served as a diagnostic adjunct to exclude alternative pathology or help predict the impact of surgery. A small number of patients were discharged from the surgical pathway because symptoms resolved, they declined further intervention, or comorbidities precluded surgery. Importantly, where patients were not adequately worked up for surgery or did not feel prepared to proceed when reviewed in the check-and-consent clinic, a DoSC was thereby avoided. One patient passed away before undergoing surgery, and one patient’s operation was cancelled in March 2023, with no procedure undertaken by study closure. Overall, these outcomes confirm that management changes made in the clinic were enacted safely, with appropriate follow-up, and without any patients being lost to care.

## Discussion

This service evaluation demonstrates that a dedicated, surgeon-led check-and-consent clinic is an effective strategy for reducing potential DoSCs in elective hand surgery. By re-evaluating patients, ahead of surgery, we identified that 43.9% required a change to their management plan. Notably, 30.3% of attendees required either a modification to their surgical plan or a shift to nonoperative management. About 12.1% of patients received no treatment at all due to symptom resolution or medical comorbidities, etc. The clinic prevented 20 almost-certain potential DoSCs and resulted in a substantial net financial saving. The high rate of management change at our clinic is reflective of the dynamic of long-term surgical decision-making in the case of common hand disorders. The finding that nearly a third of the patients no longer needed the planned operation is consistent with the known natural history of diseases that change and often respond favorably to nonsurgical management [17]. This is also consistent with the value-based healthcare principles, where only the patient with a clear necessity and preference undergoes the operation and contributes to the prevention of low-value interventions [18]. Value-based healthcare focuses on delivering treatments that offer meaningful benefits to patients while avoiding interventions that add little improvement to outcomes.

While traditional pre-op clinics excel at ensuring medical fitness and administrative readiness [10,11], the surgeon-led model used in the check-and-consent clinic directly tackles the issues of surgical appropriateness and the validity of consent, which are leading, preventable causes of cancellation [6,7]. The ethical imperative of the intervention’s clinic model is a key strength. The GMC states that doctors must "give patients the information they want or need to make decisions" and "make sure that their consent is ... given before the procedure starts" [13]. The RCS further emphasizes that consent is a process of supported decision-making, not a form to be signed [14]. The check-and-consent clinic operationalizes these guidelines by ensuring that the consent is not only informed but also contemporaneous and valid, thereby upholding the highest standards of patient-centered care.

Previous work has shown that cancelled operations impose financial burdens on the NHS running into millions of pounds annually due to wasted theater time and resources [16,19]. The economic benefits of the check-and-consent clinic extend beyond the direct savings captured in this analysis. By preventing a minimum of 20 certain DoSCs, the clinic effectively freed the equivalent of four full elective hand surgery lists, allowing theater capacity to be reallocated to other patients. From a service delivery perspective, this represents a conservative estimate, as the analysis does not capture the additional value of reduced waiting list pressure [19], improved scheduling predictability, and better patient experience. Across plausible scenarios, the clinic was consistently cost-saving, yielding net financial benefits of approximately £9,000-£14,000.

An additional consideration is the medicolegal aspect of consent. When consent is taken very close to the time of surgery, there is a possibility that patients may feel rushed or less prepared to make an informed decision. By reviewing consent ahead of the operation, the check-and-consent clinic provides patients with more time to reflect and ask questions. This may help reduce misunderstandings and support clearer documentation of the discussion, which is increasingly emphasized in national guidance.

This study has several limitations. It is a single-center, retrospective design, and the absence of a formal control group limits the generalizability of the findings. The sample size, though substantial for a focused service evaluation, is modest. Patient-reported outcomes or satisfaction measures were not captured, which would have enriched the analysis. The cost analysis is a rudimentary estimate based on national tariffs and does not account for potential downstream costs (e.g., future surgery for those postponed) or the benefits of reallocating freed theater time. Finally, the clinic was led by a single surgeon, which may introduce bias, though it also ensures consistency in the decision-making process. As this was a descriptive single-center review, the findings should be interpreted as indicative rather than definitive, and further evaluation - ideally with a controlled or prospective design - would help determine the wider applicability of this approach.

## Conclusions

The implementation of a check-and-consent clinic for elective hand surgery proved to be highly effective within the context of this service evaluation. It significantly reduced potential DoSCs by proactively identifying patients whose clinical condition or preferences had changed during the wait for surgery. This approach not only improves operating theater efficiency and generates cost savings but also firmly embeds the modern ethical principles of supported decision-making and valid consent into clinical practice. By combining cost savings with the principles of value-based healthcare, alongside the ethical imperative of maintaining valid, contemporaneous consent, the check-and-consent clinic offers a rare example of a service innovation that improves patient-centered care while simultaneously enhancing system efficiency and reducing costs. Other elective surgical services facing long waiting lists might consider adopting this simple yet powerful strategy. A prospective, controlled study comparing cancellation rates before and after the implementation of such a clinic would provide more robust evidence. Furthermore, integrating patient-reported experience measures (PREMs) would offer valuable insights into the patient's perspective on this process.

## Appendices

Appendix 1 

**Table 4:** Coding dictionary DoSC: Day-of-surgery cancellations; CTR: Carpal tunnel release.

Free-text term(s) documented in clinic letter	Classification category	Notes
Cancelled; abandoned; discharged; patient asymptomatic; spontaneous resolution; under care of another surgeon; medically unfit; patient passed away	No treatment	Counted as avoided DoSC
Injection; trial of injection; corticosteroid injection; splinting; physiotherapy; converted to conservative care	Nonoperative management	Includes trials of diagnostic or therapeutic steroid injections, even if later escalated
Changed from open to endoscopic CTR; changed laterality; unilateral ↔ bilateral; staged bilateral procedures; altered Dupuytren’s fasciectomy technique	Modified surgical plan	Procedure altered, but surgery still planned
No change documented	Management unchanged	Proceeded as originally listed

Appendix 2 

**Table 5:** Exemplar redacted clinical notes

Raw clinic note (redacted)	Classification applied
“Cancelled — spontaneous resolution”	No treatment
“Postponed — trial of steroid injection and night splinting”	Nonoperative
“Changed from open unilateral to bilateral endoscopic CTR”	Altered surgical plan

Appendix: Anonymized dataset availability

An anonymized patient-level dataset (CSV format) containing age, gender, diagnosis, initial management, final management, and coded outcome is held securely by the study team. Patient identifiers were removed, and pelvic inflammatory diseases (PIDs) were replaced with anonymized study IDs. The dataset can be made available to journal editors or reviewers upon reasonable request, in accordance with NHS trust governance and data protection policies.
